# Exploration of oxygen-mediated disinfection of medical devices reveals a high sensitivity of *Pseudomonas aeruginosa* to elevated oxygen levels

**DOI:** 10.1038/s41598-022-23082-3

**Published:** 2022-10-29

**Authors:** Francis M. Cavallo, Richard Kommers, Alexander W. Friedrich, Corinna Glasner, Jan Maarten van Dijl

**Affiliations:** 1 Department of Medical Microbiology and Infection Prevention, University of Groningen, University Medical Center Groningen, HPC EB80, Hanzeplein 1, 9713 GZ Groningen, The Netherlands; 2 Department of Medical Microbiology, University of Groningen, University Medical Center Groningen, Hanzeplein 1, P.O. Box 30001, 9700 RB Groningen, the Netherlands

**Keywords:** Microbiology, Bacteria, Bacteriology, Pathogens, Infectious diseases, Bacterial infection

## Abstract

The microbiological safety of medical devices is of paramount importance for patients and manufacturers alike. However, during usage medical devices will inevitably become contaminated with microorganisms, including opportunistic pathogens. This is a particular problem if these devices come in contact with body sites that carry high bacterial loads, such as the oral cavity. In the present study, we investigated whether high oxygen concentrations can be applied to disinfect surfaces contaminated with different Gram-positive and Gram-negative bacteria. We show that some opportunistic pathogens, exemplified by *Pseudomonas aeruginosa*, are particularly sensitive to oxygen concentrations above the atmospheric oxygen concentration of 21%. Our observations also show that high oxygen concentrations can be applied to reduce the load of *P. aeruginosa* on nebulizers that are used by cystic fibrosis patients, who are particularly susceptible to colonization and infection by this bacterium. We conclude that the efficacy of oxygen-mediated disinfection depends on the bacterial species, duration of oxygen exposure and the oxygen concentration. We consider these observations relevant, because gas mixtures with high oxygen content can be readily applied for microbial decontamination. However, the main challenge for oxygen-based disinfection approaches resides in a potentially incomplete elimination of microbial contaminants, which makes combined usage with other disinfectants like ethanol or hydrogen peroxide recommendable.

## Introduction

The elimination of potentially pathogenic microorganisms from medical equipment through disinfection or sterilization is a crucial requirement that must be addressed by manufacturers to ensure patient safety and compliance with health authority standards. According to the Centers of Disease Control and Prevention (CDC), sterilization is defined as ‘the complete elimination or destruction of all forms of microbial life, which is accomplished in health care facilities by either physical or chemical means’. Thus, sterilization is not to be confounded with disinfection, which is defined as ‘a process that eliminates many or all microorganisms, with the exception of bacterial spores’^1,2^.

There are multiple methods for disinfection or sterilization that, in daily practice, are applied based on the necessity to eliminate microbial contaminants and the properties of the devices that need to be decontaminated^2,3^. Ozone gas is currently applied as a viable alternative to conventional disinfectants, being particularly effective in those settings where the use of liquid disinfectants may prove incompatible with certain biomaterials^4^. Other common disinfection methods rely on the use of other oxidative agents, such as sodium hypochlorite, povidone iodine, hydrogen peroxide or peracetic acid^5^. Alternative options rely on the use of alcohol, chlorhexidine, quaternary ammonium compounds or glutaraldehyde^5^. In order to achieve the complete elimination of spores, autoclaving, ethylene oxide, hydrogen peroxide vapours, or plasma are preferred^5,6^. In particular, vaporized hydrogen peroxide is extensively used for the sterilization of medical devices, representing an important pillar for non-thermal gaseous sterilization approaches^7^.

The ‘Spaulding classification’ aids in the selection of appropriate levels of microbiological decontamination, which is particularly helpful for re-usable medical devices^8^. The infection risk for the patient using a medical device determines the selection of an appropriate procedure for decontamination. Specifically, Spaulding defined three different classifications for medical devices, namely critical, semi-critical and non-critical. The critical medical devices include equipment entities that enter or are in contact with sterile tissues, semi-critical devices include equipment that comes in contact with skin or membranes without penetrating them, and finally the non-critical devices include equipment that only touches intact skin but not mucous membranes^8^. These three categories are attributed in accordance with the severity of infection risk^8,9^.

A clinical condition that makes patients particularly vulnerable to microbial colonization and opportunistic infection is cystic fibrosis (CF), an inheritable disease that causes the abnormal accumulation of mucus in the lungs due to mutations in the CF transmembrane conductance regulator protein^10,11^. As a consequence, CF patients have an enhanced predisposition to airway colonization and infection by opportunistic bacterial pathogens. An example of a pathogen that takes advantage of the mucus accumulation in the airways of CF patients is *Pseudomonas aeruginosa*^12^. This bacterium is a non-sporulating aerobic pathogen of particular clinical relevance, responsible for a range of respiratory, urinary tract and surgical site infections, and bacteraemia^13^. The treatment of CF patients afflicted by *P. aeruginosa* relies on the inhalation of antibiotics, such as colistin, tobramycin, aztreonam or levofloxacine^14^. Since long-term administration of antibiotics may elicit bacterial resistance, it is important to minimize the patient’s exposure to *P. aeruginosa*. Accordingly, there is a need for simple procedures to remove this pathogen from inhalation devices that CF patients use on a daily basis. Furthermore, devices such as nebulizers are generally used at home, which calls for user-friendly decontamination protocols that do not involve toxic reagents or complicated equipment. Previous research has shown that this can be achieved by ozone treatment for merely 5 min^15^.

A potentially attractive disinfection procedure for home-use could be based on the bacterial exposure to reactive oxygen species (ROS). These ROS include highly reactive radicals, peroxides and superoxides derived from molecular oxygen (O_2_), which inflict lethal damage to bacterial cells^16,17^. The bactericidal effects of ROS are, however, only manifest if there is an imbalance between oxygen/ROS exposure and the bacterial antioxidant defences^18,19^. For instance, many bacteria are able to mitigate the destructive effects of oxygen and ROS by deploying specific enzymes, such as catalases, peroxidases and superoxide dismutases^17^. The extent to which oxygen and ROS are harmful for bacteria usually depends on the oxygen levels in their ecological niche. Thus, a broad distinction can be made between aerobes and anaerobes, with the former capable of fielding antioxidant enzymes, while the latter lack this ability. Oxygen is generally toxic for anaerobes as exemplified by strict anaerobes that can tolerate a maximum of 0.5% oxygen, while moderate obligate anaerobes can withstand a 2–8% oxygen^20^. On the other hand, the presence of antioxidants and ROS scavenging enzymes confers to aerobes a tolerance to atmospheric (21%) oxygen. Importantly, the homeostatic oxidant/antioxidant balance can be broken through an overexposure to oxygen and ROS that overwhelms the bacterial defence mechanisms and leads to bacterial death^21^.

The scope of the present study was to investigate whether oxygen-based treatments can effectively eliminate bacteria from medical devices that CF patients use at home, and to compare their efficacy to that of commonly applied disinfectants, such as ethanol and hydrogen peroxide. To this end, gas mixtures with an oxygen content higher than 21% were examined for the potential decontamination of nebulizers used to treat CF patients.

## Materials and methods

### Bacteria and growth conditions

For the present proof-of-principle study, we applied well-characterized and readily available bacterial type strains to facilitate inter-laboratory comparisons. In particular, the following strains were used throughout the experiments: *Escherichia coli* ATCC 25,922, *Staphylococcus aureus* HG-001, *Enterococcus faecalis* ATCC 29,212, *Enterococcus faecalis* ATCC 51,299, *Klebsiella pneumoniae* ATCC 11,228 and *P. aeruginosa* ATCC 27,853. Each strain was stored in 20% glycerol and frozen at -80 °C. Starting from the frozen stocks, each strain was plated on blood agar (BA) plates and grown overnight at 37 °C. The following day, 5–6 colonies were selected to inoculate 20 ml of lysogeny broth (LB; Oxoid). 100 ml glass bottles were used to culture the bacteria at 37 °C with 250 rpm shaking for 4 h. Appropriate dilutions were performed to obtain a final starting inoculum with a measured optical density at 600 nm (OD_600_) of 0.05. Of note, we applied OD_600_ measurements as a standard for the bacterial cell density throughout the present studies, instead of the alternative McFarland standard^22,23^.

### Bacterial oxygen susceptibility assay

**Figure 1:**
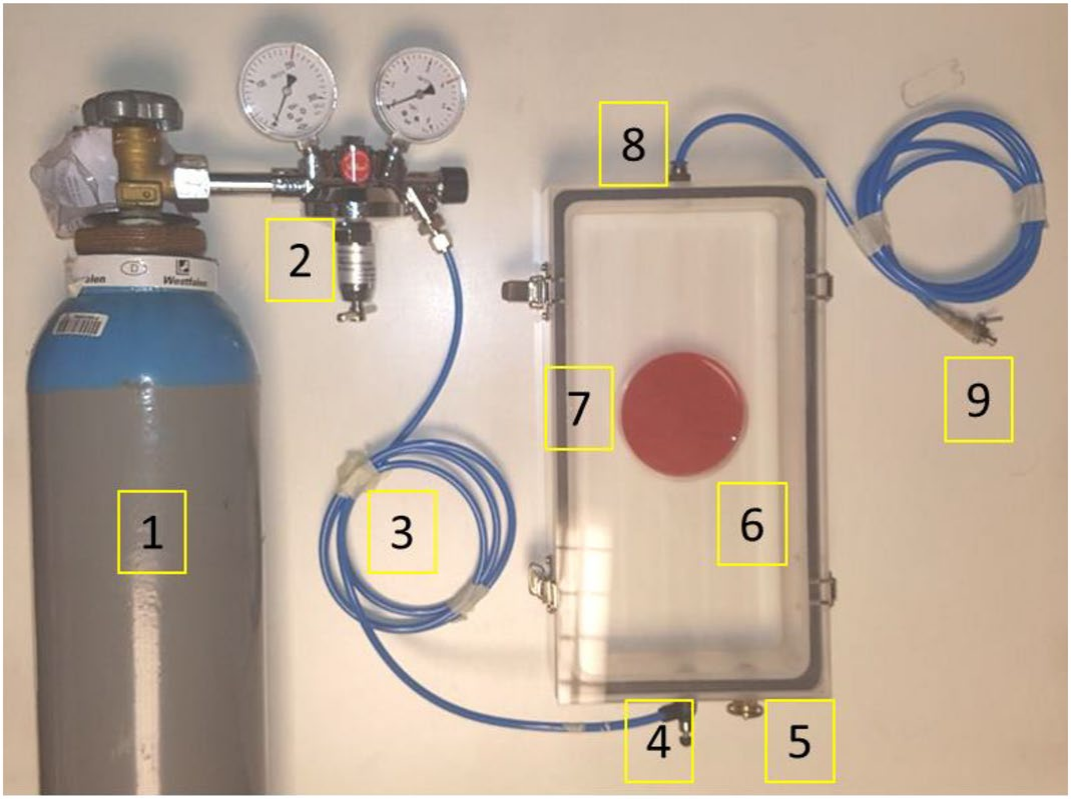
Experimental setup of gas incubation chambers. Starting from (**1**), the gas cylinder is connected to a pressure valve (**2**). A plastic tube (**3**) connects the pressure valve to the incubator inlet (**4**). On the incubator, also a pressure valve is present (**5**). Within the incubator (**6**) the experimental agar plate (**7**) or 24-well microtiter plate (not shown) is placed. Once the incubator is sealed with the lid and flushed with the appropriate gas mixture, the gas flow is allowed through an outlet (**8**) located on the polar opposite of the inlet. Finally, an adjustable tube is connected to the outlet that can be closed (**9**).

The different bacteria were grown as described above. A 1 × phosphate-buffered saline (PBS) culture suspension, corresponding to 2–4 × 10^6^ colony-forming units (CFU)/mL was selected as starting inoculum for the assay. Using 24-well plates, triplicates consisting of 20 µL of inoculum were spread across the bottom of individual wells. The plate was then allowed to air-dry, with the lid off for 1.5 h in a laminar air flow (LAF) cabinet. After drying, the plate was transferred to a custom-built gas incubator (Fig. 1). The incubator lid was closed on the chamber, and subsequently flushed for 5 min with the selected oxygen mixture at 0.8 kPa. Subsequently, the gas flow was stopped, and both the inlet and outlet of the incubator were closed. Treatment times of 1, 5, 10, or 30 min at room temperature (RT) were used. After the treatment, the incubators were opened, and the 24-well plates were incubated at RT for 20 h. Each well was then washed with 600 µL of PBS 1x, to collect the bacteria. Lastly, the remaining viable bacteria were quantified through serial dilutions, plating on BA, and colony counting, which allowed calculation of the respective CFU/mL.

### Gas incubators and oxygen mixtures

Gas incubators as shown in Fig. 1 were manufactured by the instrument makers of the University Medical Center Groningen (UMCG; ‘Research Instrumenten-makerij’). The incubators were designed in a rectangular shape, with dimensions of 300 mm × 140 mm, and a height of 40 mm. Each incubator was designed to have an inlet and an outlet to allow the flow of gasses, and a safety pressure valve. The incubators were composed of two separate parts, the chamber and the lid. The chambers were sealed with the lids through four metal clamps located on the side of each chamber. The oxygen concentrations chosen for the experiments in the present study were: 42%, 53%, 63% and 87%, the oxygen being solely complemented with nitrogen. In addition, control experiments were done with regular air, in what follows referred to as 21% oxygen.

### Nebulizers

The Ventobra nebulizer^24^, commercialized by PARI Pharma and supplied by Westfalen A.G., was chosen as a representative medical device for the experimental oxygen-mediated disinfection process. This device is ‘semi-critical’ according to the Spaulding criteria. The Ventobra system was recently approved by the European Medicines Agency for use by CF patients (EMA/169,512/2015 Page 3/3). In our study, specific attention was given to the Tolero**®** handset for the Ventobra nebulizer, used as a target for different disinfection protocols.

### Microbial contamination and decontamination of nebulizers

**Figure 2:**
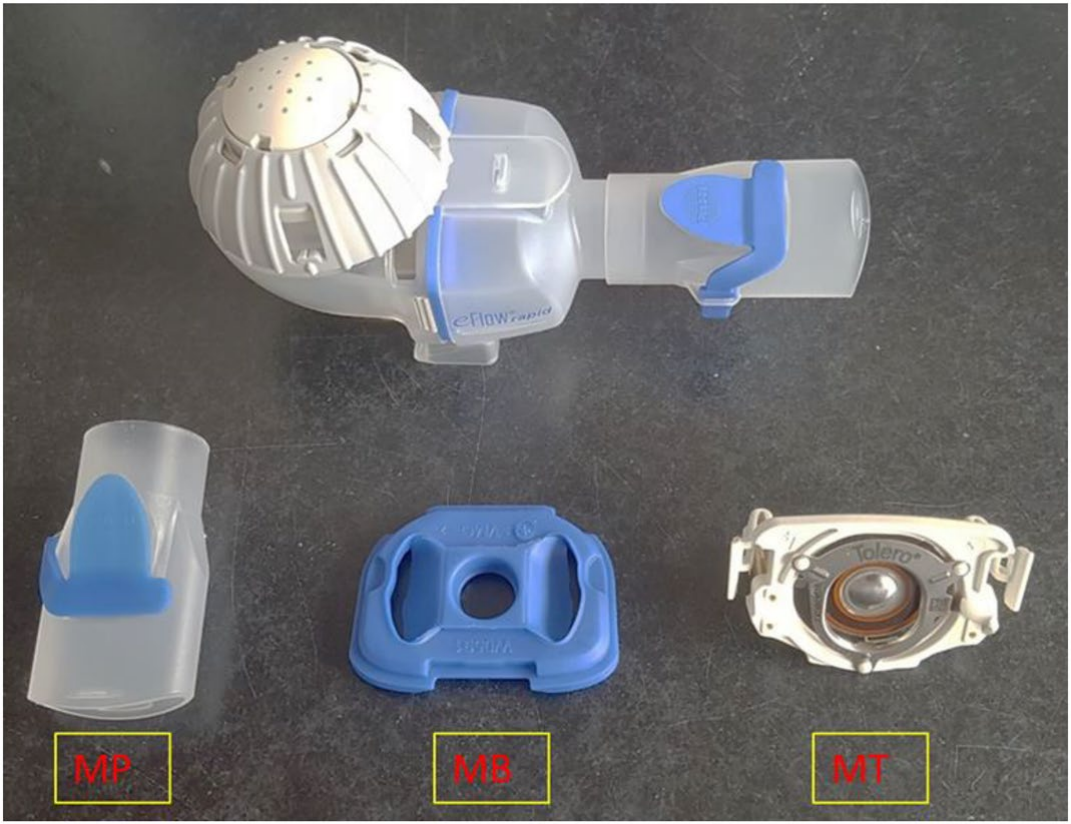
The handset of the Tolero nebulizer. The upper part of the image shows the fully assembled handset, whereas the lower part of the image shows its three main components upon disassembly, namely the mouthpiece (MP), membrane (MB), and the metal part (MT).

The Tolero handset was disassembled into its three distinct parts, namely a plastic membrane (MB), a mouth-piece (MP) and a metal piece (MT), as shown in Fig. 2. The bacterium selected for the contamination experiment was *P. aeruginosa* ATCC 27,853, grown as described above. The surfaces of the three separated device parts were purposely contaminated by bringing them into contact for ~ 10 s with a bacterial suspension of 2–4 × 10^6^ CFU/mL in PBS (OD_600_ of 0.05). The three contaminated device parts where then allowed to air-dry in a LAF cabinet and subsequently placed in gas incubators for treatment with a continuous flux of the highest oxygen concentration tested, 87% O_2_, for 30 min (0.8 kPa). After the treatment, two approaches were followed to assess the numbers of bacteria on the surfaces of the device parts. The ‘stamping’ approach entailed the contact application of each nebulizer part onto a BA plate. In contrast, the ‘swabbing’ approach entailed the collection of bacteria from the device parts’ surfaces with a sterile cotton swab and subsequent streaking of BA plates. The BA plates were then incubated overnight at 37 °C and bacterial growth was inspected on the next day.

For control, both the stamping and swabbing methods were applied in two different approaches. Firstly, the three purposely contaminated device parts were disinfected through immersion in either 70% ethanol or 30% hydrogen peroxide for 10 min, air-dried in a LAF cabinet for 10 min and tested for bacterial contamination. This allowed us to verify that the device parts were free from bacteria prior to their re-use. Secondly, to ensure that no traces of disinfectant were present on the device surfaces prior bacterial (re-)contamination, which could confound the outcomes of our experiments, the parts that had been disinfected with 70% ethanol or 30% hydrogen peroxide were immersed in sterile MilliQ water for 10 s.

### Statistical analysis

Results are presented in mean + / − standard deviation. The statistical analysis of the data was performed with GraphPad Prism 8.0.1 (GraphPad Software, USA), where a *p*-value < 0.05 was considered significant. The test applied was two-way Anova.

## Results

### Bacterial susceptibility to oxygen mixtures

**Figure 3:**
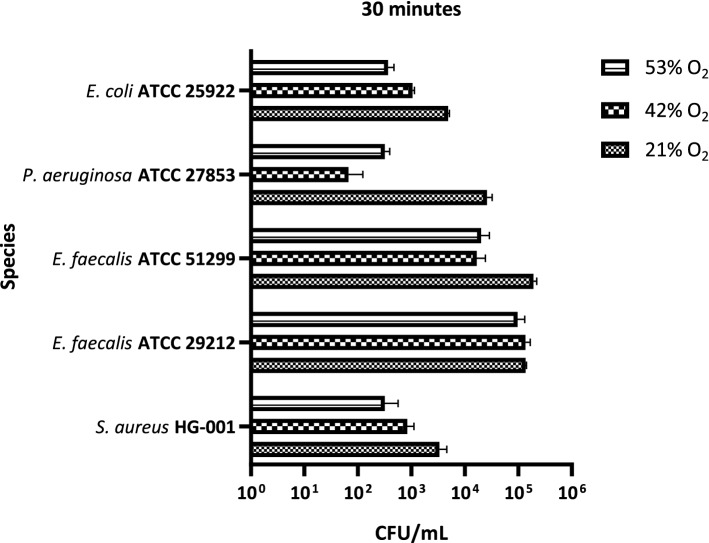
Bacterial susceptibility to oxygen. The bacterial susceptibility to oxygen is expressed in CFU/mL. The incubation time was set to 30 min at three different oxygen concentrations, namely 21%, 42% and 53%. A two-way ANOVA revealed statistically significant differences in the bacterial strain viability at increasing oxygen concentrations (*p*-value < 0.0001). Of note, we attribute the apparently slightly higher oxygen sensitivity of *P. aeruginosa* to 42% O_2_ than to 53% O_2_ to a variation in the setup of this particular series of experiments, even though the differences in the CFU/mL counts showed statistical significance.

**Figure 4:**
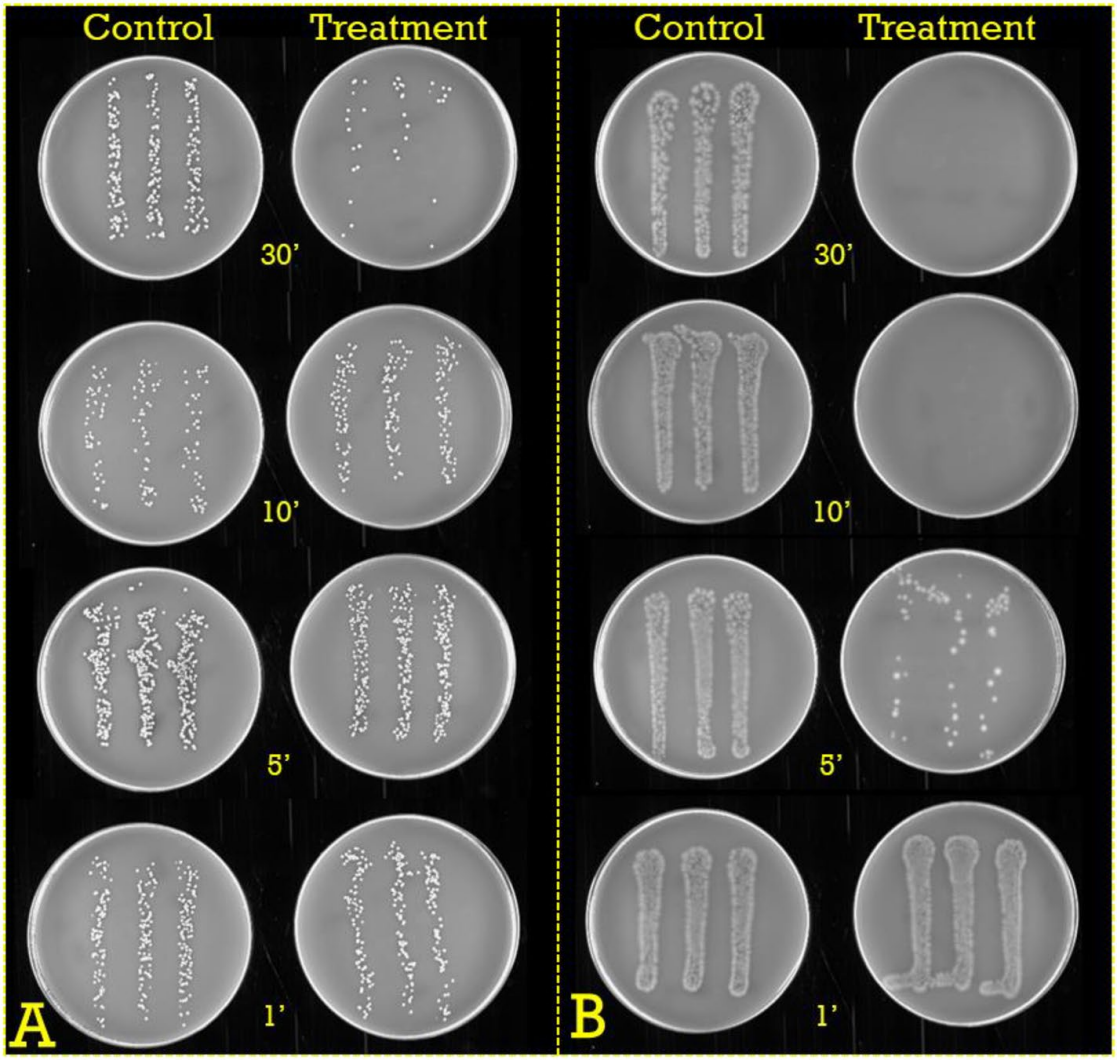
Time-dependency in oxygen-mediated killing of (**A**) *S. aureus* HG-001 and (**B**) *P. aeruginosa* ATCC 27,853. *S. aureus* HG-001 and *P. aeruginosa* ATCC 27,853 were treated with 63% O_2_ for 1, 5, 10 or 30 min, and plated on BA agar.

**Figure 5:**
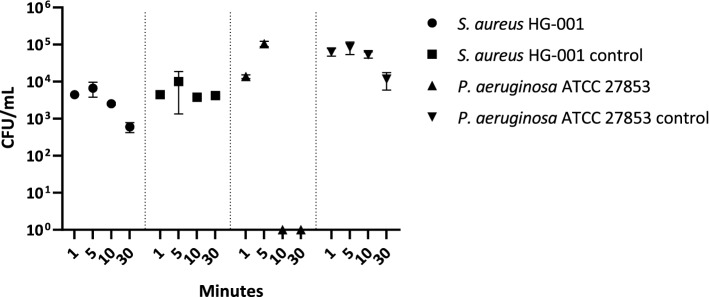
Survival of *P. aeruginosa* ATCC 27,853 and *S. aureus* HG-001 upon exposure to 63% molecular oxygen, the controls were incubated at 21% oxygen. The bacterial susceptibility to oxygen is expressed in CFU/mL. The incubation times were set to 1, 5, 10 and 30 min. A two-way ANOVA revealed a statistically significant differences in the bacterial strain viability at different time points (*p-*value < 0.0001).

The oxygen tolerance of different well-characterized bacterial type strains was investigated by exposing them in gas incubators to 21% (i.e. regular air), 43% or 53% oxygen. As presented in Figs. 3, 4 and 5 different oxygen susceptibilities were observed depending on the bacterial strain tested. In particular, upon 30 min of incubation, CFU counting indicated that *S. aureus* HG-001, *E. faecalis* ATCC 29,212, *E. faecalis* ATCC 51,299 and *E. coli* ATCC 25,922 were generally less susceptible to oxygen than *P. aeruginosa* ATCC 27,853 (Fig. 3). While the numbers of viable *S. aureus* HG-001 and *E. coli* ATCC 25,922 bacteria were reduced about tenfold at 53% oxygen, at this oxygen concentration no or at most tenfold reduction in viable counts was observed for the two *E. faecalis* strains ATCC 29,212 and ATCC 51,299, respectively. In contrast, the viable count of *P. aeruginosa* ATCC 27,853 was 500- to 100-fold reduced at oxygen concentrations above 21% (Fig. 3), suggesting that this bacterium is most susceptible to molecular oxygen exposure.

To determine whether a threefold atmospheric oxygen condition would allow more effective elimination of *S. aureus* HG-001 and *P. aeruginosa* ATCC 27,853, and to approximate the optimal incubation time, a next series of experiments was performed. As shown in Figs. 4 and 5, at 63% oxygen, the viable count of *P. aeruginosa* ATCC 27,853 rapidly decreased over time with essentially all bacteria in the inoculum being eliminated after 10 min of incubation. By contrast, *S. aureus* HG-001 demonstrated a much higher tolerance to 63% oxygen exposure, the viable count being reduced by about tenfold upon 30 min incubation.

### Nebulizer handset disinfection

**Figure 6:**
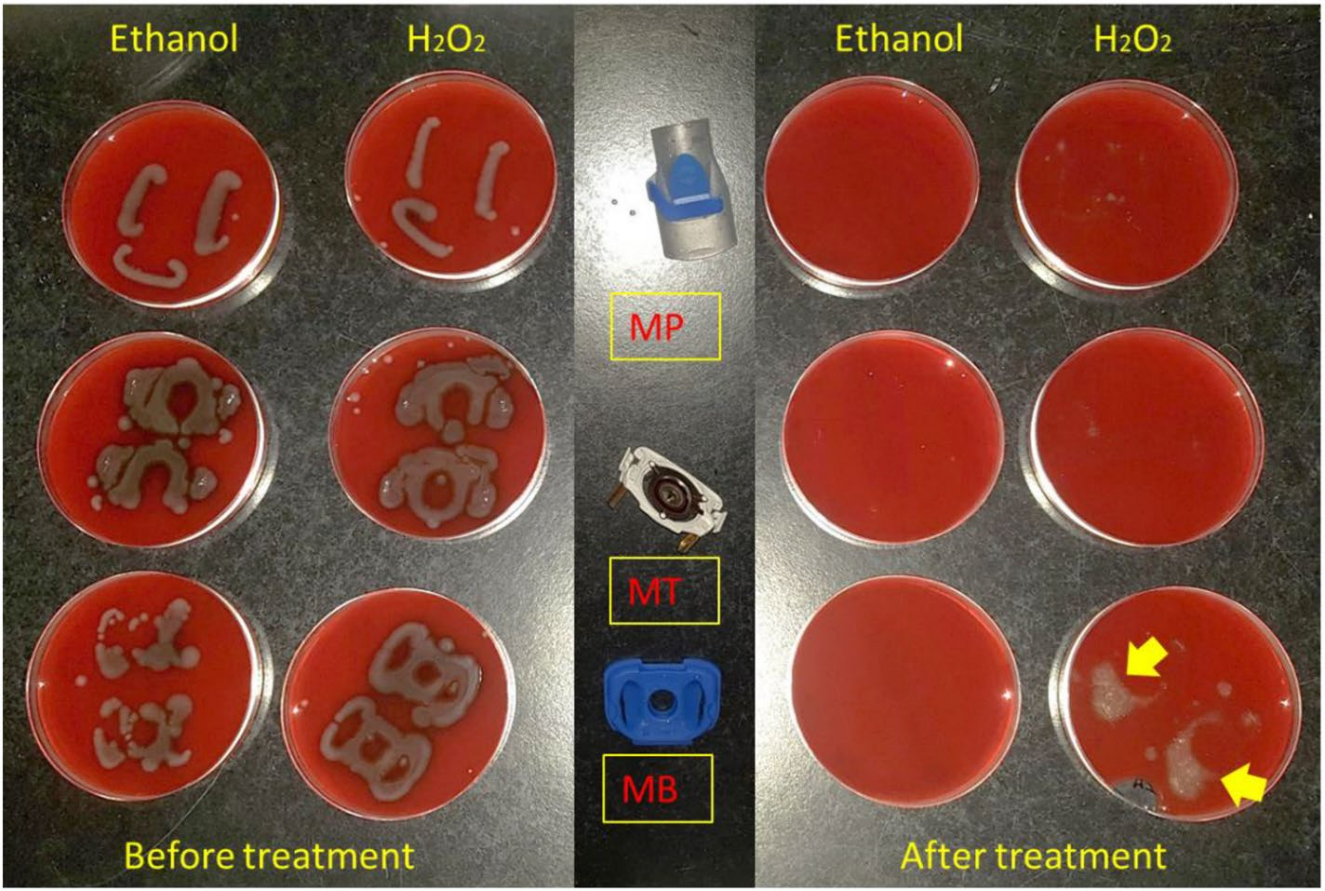
Ethanol- or hydrogen peroxide-mediated disinfection of nebulizer parts contaminated with *P. aeruginosa* ATCC 27,853. The contaminated nebulizer parts were stamped onto BA plates either before or after disinfection with 70% ethanol or 30% hydrogen peroxide (H_2_O_2_). The plates were subsequently incubated overnight at 37 °C. The yellow arrows mark hydrogen peroxide-generated foam spots. MB, membrane; MT, metal part; MP, mouthpiece.

To evaluate the possibility of disinfecting nebulizer handsets with oxygen, the Tolero handset of a Ventobra nebulizer was disassembled into its three main parts: the membrane (MB), metal part (MT) and the mouthpiece (MP). These three parts were then individually contaminated with *P. aeruginosa* ATCC 27,853. To investigate the possible oxygen-mediated disinfection, the three contaminated parts were exposed for 30 min to 87% oxygen. This condition was chosen to ensure maximal oxygen exposure. For control, the contaminated parts were disinfected with either 70% ethanol or 30% hydrogen peroxide. Upon oxygen-exposure or disinfection with ethanol or hydrogen peroxide, the different parts were ‘stamped’ onto BA plates that were subsequently incubated overnight at 37 °C. Figure 6 shows that disinfection with ethanol or hydrogen peroxide led to the complete elimination of the bacteria.

**Figure 7:**
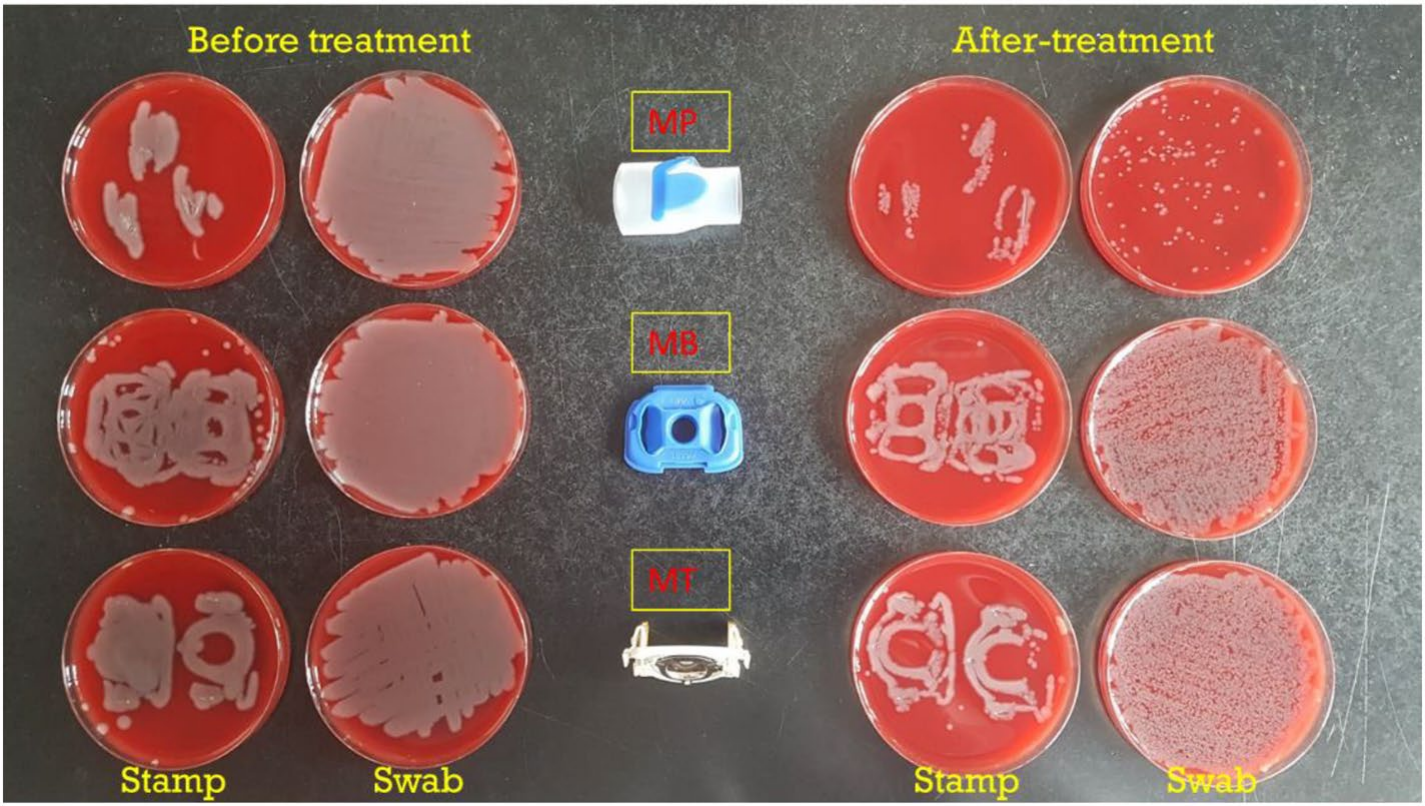
Oxygen-mediated disinfection of nebulizer parts contaminated with *P. aeruginosa* ATCC 27,853. The bacteria-contaminated nebulizer parts were pressed onto BA plates either before or after 30-min treatment with 87% oxygen. Alternatively, the deliberately bacteria-contaminated nebulizer parts were swabbed with a cotton swab stick either before or after 30-min treatment with 87% oxygen with subsequent plating of bacteria re-suspended from the swab sticks. All plates were incubated overnight at 37 °C. MB, membrane; MT, metal part; MP, mouthpiece.

Figure 7 shows the effects of 87% oxygen-mediated disinfection of the *P. aeruginosa-*contaminated nebuliser parts. Upon 30 min oxygen exposure, a clear reduction in the bacterial load was observed for all three parts, but it was particularly evident for the contaminated mouth piece. This was visualized both by the stamping and swabbing methods.

## Discussion

In the present study, we have tested the effectiveness of an oxygen-based disinfection approach against a group of opportunistic pathogens, including both Gram-negative and Gram-positive bacterial species. The bacteria were exposed to different oxygen concentrations well above the atmospheric concentration of 21%. Our results show that the susceptibility to oxygen depends on different factors, such as the bacterial species, the duration of treatment and the oxygen percentage. Interestingly, amongst the tested bacteria, *P. aeruginosa* stood out by showing the highest oxygen susceptibility. In addition, we show that one can take advantage of the oxygen sensitivity of *P. aeruginosa* to reduce its load on medical devices, as exemplified with the Tolero handset nebulizer. This handset is used by patients with pulmonary disorders like CF and, consequently, it becomes frequently contaminated with *P. aeruginosa.* While our present study shows that traditional disinfectants, such as ethanol or hydrogen peroxide, are more effective in eliminating high loads of contaminating *P. aeruginosa*, one has to realize that the high bacterial loads applied to the device parts in the present study are unlikely to be reached by daily usage. Importantly, our observations show that the most effective oxygen-mediated decontamination is achieved for the mouth piece which, during usage, is the part that will become most exposed to microorganisms in the patient’s oral cavity.

While oxygen is a potent electron acceptor that fuels the metabolism of many organisms in the three kingdoms of life, it is also a potent toxic agent. Our planet’s atmosphere contains oxygen levels of up to 21% and both aerobic microorganisms and higher organisms including humans have learned to deal with this potentially hazardous environmental condition. In general, prokaryotes are more resistant to oxygen concentrations above 40% than eukaryotes^25,26^. Thus, elevated oxygen levels can actually be applied to boost microbial growth in bioreactors, although prolonged exposure may lead to oxidative damage of both the producing microorganisms and their products^27,28^. Strikingly however, relatively little is known about the absolute limits of oxygen tolerance by different bacterial species. In particular, oxygen tolerance has been investigated in much detail for anaerobic bacteria up until the limit set by the atmospheric oxygen concentration of 21%, because higher oxygen concentrations are considered unphysiological^29,30^. For the same reason, very little attention has been attributed to the limits of oxygen tolerance by aerobic bacteria, even those that favour niches with fluctuating oxygen tensions like the human respiratory tract. This raised the question to what extents these aerobic bacteria, including opportunistic pathogens like *P. aeruginosa*, can handle oxygen concentrations higher than 21%. Accordingly, the present study aimed to explore the potentially bactericidal effects of gas mixtures, with oxygen contents higher than the atmospheric 21%, and to evaluate whether such elevated oxygen concentrations can be applied to disinfect medical devices. Our results show that *P. aeruginosa* is particularly susceptible to elevated oxygen levels. We consider this observation relevant, because *P. aeruginosa* is a notorious colonizer of the lungs of patients with impaired pulmonary functions, including CF patients, which imposes the need to minimize the bacterial loads on medical devices used by these patients.

Appropriate disinfection and sterilization of medical devices are issues of particular importance for the protection of patients with increased susceptibility for bacterial colonization and infection. Accordingly, healthcare providers and developers of medical devices have a clear need for effective protocols to eliminate or at least minimize the exposure of frail patients to potential pathogens. In addition, microbial contaminations can interfere with the functionality of medical devices. Bacterial contamination is therefore a constant issue that jeopardizes the safety of devices, particularly if they have surfaces with a relatively high water activity that promote microbial growth and come in direct contact with patients^31,32^. Another important consideration related to microbial decontamination is the reusability of expensive devices that would otherwise be discarded after use. Nebulizers belong to this category of expensive devices with a high colonization risk, which calls for effective disinfection procedures that can be applied at home. Our present findings show that this could, in principle, be achieved by exposing these devices to high oxygen concentrations in a dedicated incubator. A clear added value of oxygen-mediated disinfection would be that molecular oxygen, unlike other chemicals, will not compromise the reusability of nebulizers and other medical devices.

A possible added value derived from an oxygen-based treatment against *P. aeruginosa* would relate to the predilection of this bacterium for the mucus that accumulates in the lungs of CF patients. This mucus is depleted in oxygen, which would force the bacteria to be more relying on anaerobic respiration^33^. Interestingly, it was shown by Gupta et al*.,* that *P. aeruginosa* in anoxic conditions displays a reduced sensitivity to antibiotics, aminoglycosides in particular^34^. This observation is important, because CF patients need to undergo frequent administration of antibiotics that could, in time, become less effective due to acquired bacterial antimicrobial resistance. Potentially, disinfection of nebulizers with high oxygen concentrations could thus help to minimize the exposure of CF patients to antimicrobial resistant *P. aeruginosa*.

In conclusion, we anticipate that the toxic effects of molecular oxygen may become a powerful ally in the fight against bacterial pathogens. With the advantage of being safe for human use, oxygen represents a tool to curb the growth of particular opportunistic pathogens that contaminate and colonize biotic and abiotic surfaces. Accordingly, it could for instance be applied also in the disinfection of neonatal incubators after usage, because such incubators are expensive devices which may become contaminated with pathogens that represent a particular threat to the health of premature neonates^32^. This principle may even be extended to non-medical applications, including the food industry where elevated oxygen levels combined with low water activity may help to prevent spoilage. However, the main limitation of oxygen-based disinfection approaches resides in a potentially incomplete elimination of microbial contaminants as documented in our present study. Additional identified factors that determine the efficacy of oxygen-based disinfection are the contaminating bacterial species, the duration of oxygen exposure and the applied oxygen concentration. Until an appropriate solution is found for these potentially limiting factors, we therefore advocate approaches that combine the usage of high oxygen with other disinfectants like ethanol or hydrogen peroxide.

## Data Availability

All data generated and analysed during the current study are available.

## References

[1] Simmons BP (1983). Anticipatics, handwashing, and handwashing facililties. Am. J. Infect. Control.

[2] Rutala WA, Weber DJ, Bennett JE, Dolin R, Blaser MJ (2015). Disinfection, sterilization, and control of hospital waste. Mandell Douglas, and Bennett’s principles and practice of infectious diseases.

[3] Dempsey DJ, Thirucote RR (1988). Sterilization of medical devices: A review. J. Biomater. Appl..

[4] Rangel K (2021). Detrimental effect of ozone on pathogenic bacteria. Microorganisms.

[5] Yoo J-H (2018). Review of disinfection and sterilization—back to the basics. Infect. Chemother..

[6] Shintani H (2017). Ethylene oxide gas sterilization of medical devices. Biocontrol Sci..

[7] McEvoy B, Rowan NJ (2019). Terminal sterilization of medical devices using vaporized hydrogen peroxide: A review of current methods and emerging opportunities. J. Appl. Microbiol..

[8] Spaulding EH, Lawrence C, Block SS (1968). Chemical disinfection of medical and surgical materials. Disinfect sterilization Preserv.

[9] Mohapatra S, Prabhakar H (2017). Sterilization and disinfection. Essentials of neuroanesthesia.

[10] Scott A (2013). Cystic fibrosis. Radiol. Technol..

[11] Naehrig S, Chao C-M, Naehrlich L (2017). Cystic fibrosis. Dtsch. Aerzteblatt Online.

[12] Mulcahy LR, Isabella VM, Lewis K (2014). *Pseudomonas aeruginosa *biofilms in disease. Microb. Ecol..

[13] Lister PD, Wolter DJ, Hanson ND (2009). Antibacterial-resistant pseudomonas aeruginosa: Clinical impact and complex regulation of chromosomally encoded resistance mechanisms. Clin. Microbiol. Rev..

[14] Taccetti G (2021). Cystic fibrosis: Recent insights into inhaled antibiotic treatment and future perspectives. Antibiotics.

[15] Towle D (2018). Ozone disinfection of home nebulizers effectively kills common cystic fibrosis bacterial pathogens. Pediatr. Pulmonol..

[16] Imlay JA (2003). Pathways of oxidative damage. Annu. Rev. Microbiol..

[17] Zhao X, Drlica K (2014). Reactive oxygen species and the bacterial response to lethal stress. Curr. Opin. Microbiol..

[18] Ezraty B, Gennaris A, Barras F, Collet J-F (2017). Oxidative stress, protein damage and repair in bacteria. Nat. Rev. Microbiol..

[19] Imlay JA, Fridovich I (1991). Assay of metabolic superoxide production in Escherichia coli. J. Biol. Chem..

[20] Noor A, Khetarpal S (2021). Anaerobic infections.

[21] Buccellato LJ, Tso M, Akinci OI, Chandel NS, Budinger GRS (2004). Reactive oxygen species are required for hyperoxia-induced bax activation and cell death in alveolar epithelial cells. J. Biol. Chem..

[22] Peñuelas-Urquides K (2013). Measuring of mycobacterium tuberculosis growth: A correlation of the optical measurements with colony forming units. Braz. J. Microbiol..

[23] Kralik P, Beran V, Pavlik I (2012). Enumeration of mycobacterium avium subsp. paratuberculosis by quantitative real-time PCR, culture on solid media and optical densitometry. BMC Res. Notes.

[24] Knoch M, Keller M (2005). The customised electronic nebuliser: A new category of liquid aerosol drug delivery systems. Expert Opin. Drug Deliv..

[25] Lin AA, Miller WM (1992). Modulation of glutathione level in CHO cells. Ann. N. Y. Acad. Sci..

[26] Gille J (1988). Effect of normobaric hyperoxia on antioxidant defenses of Hela and CHO cells. Free Radic. Biol. Med..

[27] Lara AR (2011). Comparison of oxygen enriched air vs. pressure cultivations to increase oxygen transfer and to scale-up plasmid DNA production fermentations. Eng. Life Sci..

[28] Oosterhuis NMG, Kossen NWF (1984). Dissolved oxygen concentration profiles in a production-scale bioreactor. Biotechnol. Bioeng..

[29] Tally FP, Stewart PR, Sutter VL, Rosenblatt JE (1975). Oxygen tolerance of fresh clinical anaerobic bacteria. J. Clin. Microbiol..

[30] Brusa T, Canzi E, Pacini N, Zanchi R, Ferrari A (1989). Oxygen tolerance of anaerobic bacteria isolated from human feces. Curr. Microbiol..

[31] de Goffau MC, van Dijl JM, Harmsen HJM (2011). Microbial growth on the edge of desiccation. Environ. Microbiol..

[32] de Goffau MC (2011). Cold spots in neonatal incubators are hot spots for microbial contamination. Appl. Environ. Microbiol..

[33] Alvarez-Ortega C, Harwood CS (2007). Responses of *Pseudomonas aeruginosa *to low oxygen indicate that growth in the cystic fibrosis lung is by aerobic respiration. Mol. Microbiol..

[34] Gupta S, Laskar N, Kadouri DE (2016). Evaluating the effect of oxygen concentrations on antibiotic sensitivity, growth, and biofilm formation of human pathogens. Microbiol. Insights.

